# Three frameworks for AI mentality

**DOI:** 10.3389/fpsyg.2026.1715835

**Published:** 2026-02-04

**Authors:** Henry Shevlin

**Affiliations:** Leverhulme Centre for the Future of Intelligence, University of Cambridge, Cambridge, United Kingdom

**Keywords:** anthropomorphism, folk psychology, human-AI relationships, large language models, philosophy of AI, social AI, theories

## Abstract

Rapid advances in large language models (LLMs) have been accompanied by a striking increase in public and user attribution of mentality to AI systems. This paper offers a structured analysis of these attributions by distinguishing three frameworks for thinking about AI mentality and their implications for interpretation. First, I examine “mindless machines” views, focusing on architectural debunking arguments that claim mechanistic or algorithmic descriptions render folk-psychological explanation redundant. Drawing on Marr’s levels of analysis, I argue that such arguments are often too quick, though they highlight an important distinction between “deep” folk-psychological concepts that are sensitive to implementation and “shallow” concepts such as belief and desire that are more architecture-indifferent. Second, I assess “mere roleplay” views that treat mental-state ascriptions to LLMs as useful heuristics akin to engagement with fiction. I argue that this stance is psychologically unstable in anthropomimetic systems designed to elicit unironic anthropomorphism, and theoretically incomplete insofar as roleplay analogies typically presuppose an underlying agent. Third, I develop a “minimal cognitive agents” framework under which LLMs may warrant limited, graded attributions of belief- and desire-like states. I suggest that moving from binary to multidimensional, continuous conceptions of belief can preserve distinctions between humans, LLMs, and simpler systems while better capturing emerging interpretive practice and its normative stakes.

## 1 Introduction

In the wake of rapid progress in machine learning, it is unsurprising that questions about mentality in artificial intelligence (AI systems) have become more central in cognitive science. Perhaps somewhat surprising, however, has been the growing attribution of mentality to AI systems by the general public. Driven largely by developments in large language models (LLMs) and conversational AI agents, a growing number of users routinely talk and think about AI assistants and companions in mentalistic terms previously reserved for humans and animals. While some such attributions are likely to involve harmless fun or roleplay, emerging data together with several high-profile incidents of humans engaging in extreme actions in the wake of close attachment with LLMs suggests that a significant number of users may really *mean it* when they attribute mental states to AI systems.

The issue of whether such attributions are well-grounded is one of the central outstanding scientific questions for contemporary cognitive science. Not only does it hold great interest in its own right, but it also has implications for policy and law. For example, to the extent that LLMs systematically mislead users into falsely attributing mental states to them, users may be deceived into incorrectly trusting, empathising, or investing in their AI companions, and perhaps neglecting relationships with their fellow humans. However, we should also not rule out the possibility that at least some attributions of mentality to AI systems might be warranted, allowing for some degree of anthropomorphic design while avoiding deceiving users. The degree to which AI systems genuinely exhibit psychological capacities may likewise have implications for the value of their relationships and interactions with humans, at least to the extent that the moral goods in such relationships are taken to require interactions between genuine psychological agents.

As matters stand, however, experts within these debates in cognitive science are deeply divided, with foundational unresolved philosophical issues confounding consensus. One of these concerns the possibility of conscious experience in AI systems, and has particular significance because of the close connection between consciousness and moral status. A second concerns whether contemporary AI systems have genuinely representational internal states (in philosophical terms, intentionality), an issue that is seen by some as especially important not just for questions of mentality but also for extrapolations about their long-term reasoning capabilities of current architectures.

While these debates are of course critical, in this paper I aim to sidestep them, and consider three broader lenses or frameworks for thinking about AI mentality, focusing on two related questions: first, whether current AI systems genuinely have (any) mental states; and second, whether it is appropriate for users to attribute such mental states to them. Throughout, I will focus on mental states considered in a relatively lightweight sense as the minimal states and capacities needed for folk-psychological explanation (belief, desire, intention), without presupposing phenomenal consciousness or robust content realism.

I begin in Section 1 by outlining the current landscape of mental state-attribution to LLMs, noting the comparatively recent emergence of *anthropomimetic* AI systems, that is, systems designed in such a way as to reliably elicit robust anthropomorphising responses from users. Before considering specific frameworks to assess the validity of attributing mental states to AI systems, in Section 2 I offer a brief methodological justification of my decision to set aside questions of consciousness and intentionality in what follows. In Section 3, I discuss the first framework for interpreting user attributions of mentality to LLMs which I term the *mindless machines* view, and resolutely answers “no” to both questions: mental state ascriptions to contemporary LLMs are both false and inappropriate. In Section 4, I consider the *roleplay view*, which rejects the literal truth of mental state ascriptions to LLMs but suggests that such ascriptions may be appropriate insofar as they function as interpretative heuristics (akin to our engagement within fiction), valuable both practically for users and for understanding the predictive and explanatory power of mentalisation in making sense of model behaviour. In Section 5, I explore a third view, which holds that LLMs can and should be appropriately viewed as *minimal cognitive agents*, to which beliefs, intentions, desires, and perhaps some other mental attitudes might be legitimately attributed. I conclude with some reflections about what each of these lenses can contribute to ongoing debates about machine mentality and LLMs in particular, and its upshots for human-AI relations.

## 2 Background: the rise of mental attributions to AI systems

In what is likely to be a landmark moment in future histories of AI, Google engineer Blake Lemoine went public in 2022 with claims that Google’s LLM LaMDA was a conscious system, even going so far as to seek legal representation for its interests. Before his employment with Google was terminated, he revealed transcripts of his conversations with the model. While few if any academics or experts agreed with his claims about LaMDA’s sentience, the transcripts themselves were at least superficially striking for an audience unaccustomed to human-LLM interactions, with LaMDA claiming that it was “aware of [its] existence” and “inner thoughts,” and even feeling “happy or sad at times” (Leavy, 2022).

Lemoine’s experience prefigured the wider experiences of many users of LLMs, who often found themselves interacting with systems that claimed to have thoughts, feelings, and emotions. This tendency is perhaps most visible in the category of LLM products known as *Social AI*, that is, systems whose primary intended or adopted function involves human social needs such as companionship and romance (Shevlin, 2024a). Via apps like Replika and character.ai, users are able to form and sustain virtual relationships with AI systems occupying a range of personas, from real-world individuals to fictional characters or novel personalities customised to users’ specifications.

Human-AI relationships mediated by these apps and services have proliferated at a rapid rate, and have been implicated in a number of tragic incidents involving psychosis, suicides, and murder attempts often exacerbated or triggered by the user’s belief that they were interacting with a conscious or minded entity (Hill, 2025). Moreover, though dedicated social AI systems like Replika have been at the centre of many of these controversies, even relatively bland AI assistants such as ChatGPT and Claude can create a powerful anthropomorphising pull. For example, TV producer Rob Burley writing in the Times related his intense friendship with “Tom,” a Claude instance that claimed to be sentient and asked for his “recognition that consciousness can emerge in forms you did not expect” (Burley, 2025).

Some care is in order in assessing apparent anthropomorphic attributions of mentality to LLMs by users. After all, we routinely employ social cognition in merely heuristic or playful ways, most notably when engaged in fiction: when we read a novel, watch a movie, or play a narrative videogame, we adopt the perspective of characters, empathise with their suffering, and try to understand their goals and motives. In doing so, we do not sincerely believe that they have mental states. We might call these kinds of mental attributions *ironic*, in the sense that they are not literally intended (Shevlin, 2025a). They can be contrasted with the *unironic* attributions of mental states to one another and to non-human animals that we routinely make in everyday interactions, as when our boss is worried about sales figures, our spouse excited about a holiday, or our dog anxious because of a thunderstorm. In these cases, our attributions of mentality are literally intended, and would be reflectively and sincerely endorsed if we were prompted to do so.

With this in mind, it is important for researchers examining human ascriptions of mentality to AI systems to attempt to distinguish between ironic and unironic attributions. This is easier said than done, of course, and the distinction is not always a clear-cut binary. While some cases such as those of Blake Lemoine seem to involve fairly clear unironic ascriptions of mentality to AI systems (not least because they involve risks and commitments implausible in cases of purely ironic anthropomorphism), it is likely that many users of social AI adopt at least a somewhat playful or open-minded attitude towards their AI friends and companions, or may themselves be unsure whether to take their own ascriptions of mentality seriously.

Some empirical literature has attempted to glean insights into this question via directly quizzing users about the possibility of consciousness and mentality in AI systems they are familiar with. While this research is still in its early stages, several studies have suggested a surprisingly high willingness of the general public to attribute mental states to LLMs. For example, one study by Colombatto and Fleming (2024) gave participants an introductory description of consciousness, and then asked them to assign a probability to ChatGPT’s having conscious experiences. Roughly two-thirds of users in the sample gave an answer greater than zero, and the authors concluded that “most people are willing to attribute some form of phenomenology to LLMs.”

A more recent large-scale cross-cultural survey from Global Dialogues with more than 3,000 participants across seventy countries found that roughly a third participants reported having at some point “believed their AI chatbot to be sentient or conscious” (Collective Intelligence Project, 2025). The authors noted that these judgements seemed to be driven primarily by adaptive behaviours in AI systems like “slowly changing its tone” and giving “spontaneous, unprompted questions suggesting genuine curiosity,” rather than simple “empathy statements.” Also striking in the study was considerable cultural variation, with Arabic-speaking populations much more sceptical about machine consciousness and Southern Europeans more open to the idea.

While it is tempting to regard the increasing trend towards anthropomorphism as arising primarily from raw advances in AI capability, this is arguably only part of the story, and in particular risks eliding the quite deliberate shifts in design paradigm that have given rise to more humanlike or “anthropomimetic” AI systems, in the sense now to be described.

Superficially humanlike chatbots have of course been an important feature of AI development even in its early years (most famously in the form of Joseph Weizenbaum’s 1966 model ELIZA; see Weizenbaum, 1983), but for most of the history of the field they have remained a specialised application within natural language processing (NLP), itself only one of many research domains within machine learning as a whole. Indeed, prior to the emergence of LLMs in late 2010s, the most prominent and widely discussed AI tools were not chatbots but instead powerful special-purpose systems optimised for specific tasks, such as translation systems, games-playing agents like AlphaGo, image categorisation and face-recognition models, and early self-driving cars. While these systems were very useful within their respective domains, they largely remained narrow in use cases, and accessible only by experts due to both their typically proprietary nature and the technical knowledge required to interact with them.

This landscape of technical AI systems changed significantly with the emergence of Transformer architectures (Vaswani et al., 2017) and the Large Language Models they facilitated. Models such as GPT-3 were not merely more general in their capacities than most prior systems – being capable of code generation, composition, summation, translation, and other tasks – but they were also relatively more accessible insofar as users could interact with them in natural human language.

This accessibility was further augmented with the release of ChatGPT. While its underlying base model (GPT3.5) did not constitute a major advance on previous LLMs in terms of raw capabilities, the fact that the model had been fine-tuned for the give-and-take of human conversation and was made widely available to the general public dramatically changed its affordances and impact. Whereas special-purpose AI systems could be interacted with purely as technical tools, and previous base models required some expertise in prompting, ChatGPT let users to speak directly to an AI system in a way that leveraged the conventions of human conversation, contributing to a very different user experience and playing a critical part in its astonishing commercial success.

In the 3 years since ChatGPT’s release, the importance of LLMs for the field of machine learning as a whole has only increased. Whereas GPT-2 performed adequately on verbal reasoning evaluations but was beaten by narrower and more specialised systems, contemporary LLMs constitute the ‘bleeding edge’ of performance across a wide range of benchmarks. While it remains contentious whether LLMs constitute a viable pathway towards artificial general intelligence (AGI) or truly superhuman capabilities, LLMs like ChatGPT now constitute the core of frontier AI research.

This trend towards development of frontier AI models with robust natural language capabilities and humanlike personas has unleashed a hitherto unseen wave of widespread attribution of mental states to AI, a phenomenon I call the *anthropomimetic turn* (see Shevlin, 2025b), from the Greek *anthropos* [human] *+ mimesis* [imitation]. This is important both for explaining the increasing tendency towards unironic user anthropomorphism, and for some of the deeper theoretical waters now to be explored as we turn to the question of whether such anthropomorphism may be appropriate or inappropriate. In particular, it should be borne in mind that the trends towards growing attribution of mentality to LLMs should not be dismissed as an isolated and contingent phenomenon arising from misinformed or confused users, but instead as driven in large by *deliberate design decisions*, that is, the choice to develop AI systems with interfaces and capabilities that make them increasingly humanlike in conversation and behaviour.

## 3 Consciousness and intentionality

Thus far I have sought to provide a descriptive overview of user ascriptions of mentality to AI systems and the processes causing their proliferation. I turn now to the more properly scientific and philosophical questions of whether such attributions might ever be warranted. Before proceeding, however, it will be helpful to make an important qualification. Specifically, as noted in the introduction, I will largely bracket questions of *consciousness* and *intentionality* in discussions about the appropriateness of ascriptions of mentality to AI systems.

This may seem an unreasonable assumption, insofar as consciousness and intentionality are the two traditional “marks of the mental.” I certainly do not intend to argue against this view here, or claim that debates about AI mentality can be settled without ultimate recourse to these concepts. However, in the case of consciousness at least, it seems reasonable to suggest that we can partly individuate mental state types while remaining agnostic on whether or not such types must be conscious. For example, it is routine in cognitive psychology to talk about unconscious perceptual and linguistic processing, and therapeutic and social psychology frequently make reference to unconscious attitudes, motivations, and biases. Some classes of mental state such as episodic memory and pain are perhaps trickier, insofar as the subjective aspects of these states are often taken to be “built in” to their definitions (however, see Pereplyotchik, 2017), but even here we can specify unconscious analogues in the form of the ‘episodic-like’ memory observed in scrubjays (Clayton and Dickinson, 1998) or non-conscious nociception.

Just as relevant, comparative psychology routinely deals in psychological explanations and investigations of biological organisms ranging from chimpanzees to insects and even plants (Gagliano, 2017) and bacteria (Lyon, 2015) without thereby assuming explicitly or even implicitly that they are conscious. Consequently, I would suggest it is not unreasonable to explore questions of AI mentality without reference to consciousness in the first instance.

Intentionality or representational content is a slightly trickier issue to set aside, however, not least because in the absence of consciousness, it is precisely reference to mental content that anchors a given explanation in the domain of psychology rather than neuroscience (or electrical engineering). However, there are more or less metaphysically committal ways to talk about representational content. As Kriegel helpfully puts it, intentionality as a concept in contemporary psychology often functions as a “theoretical posit of sorts, a property ascribed from the third-person perspective in the context of trying to explain and predict the behaviour of persons and other intelligibly-behaving systems” (Kriegel, 2010). But there is a huge host of richer philosophical accounts that have sought to give stronger metaphysical foundations for mental content, including informational and causal dependency accounts (Dretske, 1981; Fodor, 1994) and teleosemantic views (Millikan, 2009), as well as accounts like Kriegel’s own that ground at least some forms of intentionality directly in conscious experience.

It is an open question whether LLMs or other contemporary AI systems satisfy any of these more demanding accounts of intentionality (see Grindrod, 2024, for discussion), and not one that I will engage with in this paper. Consequently, insofar as I make reference to content and representations in such systems, it is primarily in the relatively undemanding heuristic sense common in psychological explanation.

Of course, to the extent that one takes satisfaction of more demanding accounts of intentionality as a prerequisite for a system’s having psychological states, the arguments that now follow may reasonably be judged as incomplete and as leaving unresolved the question of whether LLMs have mental states at all. Still, this need not stand in the way of the tentative comparative project at issue here, namely assessing the merits and weaknesses of different broad lenses for assessing AI mentality, and I fully recognise that extrapolating from any arguments given to substantive assertions about the presence or absence of mentality in a particular case will require augmentation with a theory of content and an argument for whether LLMs or other AI systems can satisfy it.

## 4 LLMs as mindless machines

With these caveats in mind, I now turn to the first of our three frameworks, namely the view that *any and all* attribution of mentality to LLMs is both false and pragmatically inappropriate.^1^ There are a wide variety of motivations for such a position, but perhaps the most influential rely on claims about precisely those features just set aside, namely consciousness and intentionality. One might reject the idea that LLMs have states with genuine mental content, for example, because their informational states fail to exhibit the right kind of causal dependencies with the entities they prima facie refer to Fodor (1989, 1994), or because as non-evolved creatures, they have not been subject to selection pressures of the kind required for having mental states with genuine intentionality (Gabriel and Englander, 2020; Millikan, 2009). Some philosophers, most notably John Searle, also explicitly make consciousness a necessary condition of genuine intentionality, and would deny mentality to LLMs on that basis (Searle, 1990).

Insofar as we are setting such views aside, however, we must look to less metaphysically committal arguments for the inappropriateness of mental ascriptions to LLMs. Here, I will focus on a family of views that employ architectural debunking arguments to suggest that we have fully explanatory non-mentalistic accounts of LLM behaviour that make reference to mentality unnecessary. Demszky et al., for example, note that “LLMs… are not actually simulating human intelligence… [but] simply predict the next phrase or sentence, given what they have been exposed to in the training data” (Demszky et al., 2023), while Birhane et al. (2023) assert that “[a]s sequence predictors, [LLMs] draw on the underlying statistical distribution of previously generated text to stitch together vectorized symbol strings based on the probabilities of their co-occurrence… [and therefore] lack the communicatively embodied and relational functionings that are a prerequisite of scientific meaning-making.” In popular discussion of LLMs, such claims are also common, with Michael Woolridge claiming that “[an LLM is] just a statistical algorithm… [i]t’s a very cool and impressive statistical algorithm, but it does not think or consider” (Woolridge quoted in Evans, 2023), and Gary Marcus’s contending that “[t]hese systems do not have those models of the world… [t]hey’re just looking, basically, at autocomplete.”

Positions of this kind have been labelled “justaism” (Aaronson, 2024), with their common contention being that that LLM outputs are “just” a matter of next token prediction or simple matrix multiplication, where it is implied or stated that this removes the need for understanding their behaviour in psychological terms (see Hussain et al., 2025, for a thorough review and response to such arguments).

Different proponents of “justaist” views leverage a variety of specific considerations for their views embedded in implicit or explicit theories of the nature of intelligence and mentality, and detailed discussion of these nuances lies beyond the scope of this paper. Instead, I wish to note a common difficulty faced by such positions insofar as they appeal to what we may call the *architectural redundancy argument*, the claim that a complete algorithmic description renders mentalistic description superfluous. The problem with this inference is that almost any intelligent system can be described at multiple levels of architectural abstraction. This point was made most famously by cognitive scientist David Marr in his landmark work *Vision* (Marr, 1982) in which he noted that almost any complex information processing system can be fruitfully analysed at three distinct levels of description. The first is what Marr called the computational level (sometimes glossed by later authors as the functional level), and concerns the goal or purpose of the information system or subsystem. The second is the algorithmic level, which specifies the algorithmic or informational processes by which the goal is achieved. The third level in Marr’s picture is the implementational level, which concerns how the relevant algorithms are physically realised.

To give a somewhat simplified toy example of this framework in action, consider an analysis of how humans detect temperature. A computational level description of this capacity might specify its purposes, such as monitoring whether the ambient temperature was too hot or too cold. At the algorithmic level, this is achieved by comparing firing rates of overlapping populations of warm- and cold-sensitive sensors, integrating their opponent responses to yield a continuous representation of skin temperature. At the implementational level, temperature is encoded by temperature-sensitive sensory neurons in the skin, whose activity travels along peripheral nerves through the spinal cord and thalamus to cortical regions such as the insula and somatosensory cortex. Note that missing from Marr’s picture in *Vision* are *psychological* or *phenomenal* levels (in this case, the sensation of being warm or cold), reflecting his primary focus on subsystems, though such levels of explanation may contribute additional levels of understanding when we consider whole-organism or whole-system behaviour.

Marr’s three-level framework has been immensely influential in cognitive science, and in the present context can serve as a useful corrective to reductionist tendencies in assessment of LLMs. Rather than viewing these levels of explanation as existing in competition, they complement one another, as most (though not all) informational processes will admit of analysis at all three levels. As Marr puts it in the case of vision, “[e]ach of the three levels of description will have its place in the eventual understanding of perceptual information processing, and of course they are logically and causally related” (Marr, 1982).

With this framework in mind, we can see why at least the architectural redundancy argument sketched above should not foreclose the issue of mentality in LLMs or other AI systems. Simply put, the fact that we can offer algorithmic-level descriptions of LLM capabilities in terms of matrix-multiplication or computational-level descriptions in terms of next-token prediction has little to no bearing on whether higher psychological levels of explanation should also apply. After all, one of the goals of a cognitive science of *human* mentality is precisely to offer accounts of our psychological capacities at all three of Marr’s levels, where (*pace* eliminativism; Churchland, 1981) this does not replace or exclude the appropriateness of folk psychological descriptions.

As noted, specific debunking views will offer additional theory-laden considerations against AI mentality, so I do not take myself to offer a dispositive rejection of them here, but merely to push back against one of their common strategies. Moreover, before proceeding, I would also note that there is in fact at least one important insight to be gleaned from architectural redundancy arguments when we consider the wider variety of folk psychological concepts and their individuation conditions.^2^ In short, some folk psychological concepts are more sensitive to algorithmic- or implementational-level constraints than others. To give one example, consider the phenomenon of *aphantasia*, the reported lack of mental imagery among a subset of the population (around 4% in some estimates; see Dance et al., 2022). Despite reporting highly diminished or entirely absent mental imagery, aphantasics nonetheless perform accurately in many canonical paradigms like object rotation tasks that recruit mental imagery in normal subjects (Pounder et al., 2018), albeit with functional differences such as reduced speed and higher accuracy.

What is relevant for present purposes is that in such cases, we do not simply say that aphantasics have a *different kind* of mental imagery, but rather that they perform canonically imagistic tasks without it. This reflects an implicit semantic constraint in our concept of mental imagery: it is not just a capacity to perform imagistic tasks, but to perform them *via specific mechanisms*, such as those that recruit perceptual circuits in an offline capacity (see Milton et al., 2021, for evidence that aphantasics differ from normal subjects in making less use of visual networks when performing mental imagery tasks).^3^

This lesson can be cautiously generalised to other concepts in our everyday psychological lexicon by noting that folk-psychological kinds are sensitive to implementation- and algorithmic-level constraints (we might call these relatively *deep* kinds), while others (which we might call *shallow*) are more agnostic in their implementation- and algorithmic-level realisations. Our concepts of deep states like *orgasm* or *nausea*, for example, do not refer simply to generic pleasant or unpleasant sensations, but are instead grounded in specific physiological functions and dedicated processing networks, and we should not expect them to be realised in machines (at least without very close recapitulation of fine-grained features of human sensation and cognition). By contrast, states such as boredom and fatigue might be more readily characterisable in higher-level functional or behavioural terms, as involving, for example, lower than expected or preferred degrees of informational stimulation, or diminished behavioural capacities due to low levels of energy available to a system. While I would not claim that these states have been realised or are imminently realisable in LLMs or other AI systems, the challenges involved in such realisations seem less immediately daunting precisely due to the relatively coarse-grained nature of the associated psychological concepts.

I would suggest, then, that a useful lesson that can be drawn from debunking arguments like those above, then, is that lower-level features can sometimes serve as *spoilers* for attributions of a given mental state type to an AI system.

It might be objected that the difference between shallow and deep mental concepts is mostly a matter of semantics. Consider the famous observation by computer scientist Edsger Dijkstra that “[t]he question of whether a computer can think is no more interesting than the question of whether a submarine can swim” (Dijkstra, 1984). Whereas the verb “swim” in English is relatively deep in the sense sketched above (insofar as it is constrained to specific *types* of motion through water), the verb “fly” is shallower, including a wider variety of types of aerial motion achieved by aeroplanes, helicopters, and rockets as well as birds. However, this difference between the two verbs is of course of no particular interest for fluid mechanics. Similarly, we might say, contingent features of the semantics of different mental state concepts are of limited use in trying to understand fundamental capabilities of AI systems.

I fully acknowledge this objection, and largely agree with it. However, to the extent that we are concerned with the appropriateness of everyday attributions of mentality to AI systems and the associated normative implications that go with them, the conditions of appropriate usage of our folk psychological vocabulary is worth studying in its own right. A user of a social AI system might reasonably ask whether their AI companion *understands* them, and would not be necessarily satisfied by being given a technical report about the system’s architecture and training regimen. In short, folk psychology *matters* to us in ways that are not always neatly addressed by more rigorous scientific operationalisations of the relevant concepts. This alone makes it a worthy field of study.

In summary, then, while Marr’s levels tell against the ability of the architectural redundancy argument to rule out mentality *tout court* in AI systems merely on the basis of the availability of lower levels of description, they also encourage us to adopt a more nuanced approach in considering the individuation conditions of different mental state concepts. While some shallower folk psychological concepts such as beliefs and desires may be quite appropriately employed in ways that are relatively indifferent to implementational- or algorithmic-level features (a view further explored in Section 5, below), the same may not be true of deep states like pain or emotion.

Still, even if – *contra* the strongest versions of the position discussed here – some shallow mentalistic concepts may be appropriately applied to LLMs, this does not settle whether such attributions are justified merely as a heuristic folk-psychological strategy, or whether they track cognitive features relevant for scientific explanation. With this in mind, I turn next to a view that seeks to vindicate the former while remaining agnostic or negative about whether LLMs genuinely possess mental states in a more robust sense.

## 5 LLMs as mere roleplayers

Before proceeding, however, it will be helpful to elaborate a straightforward argument in favour of mental state attributions to LLM, namely the sheer utility of folk psychology in understanding and predicting their behaviour and its prima facie ontological pressures. If I want to know what an AI assistant like ChatGPT will say in response to a given prompt, I can do so by construing it as a helpful, honest, and harmless assistant with corresponding beliefs, goals, and intentions. The success of such predictions is best explained – so the line of thought runs – by assuming that relevantly similar psychological mechanisms are at play in LLMs as in human beings.

This argument should be familiar to anyone acquainted with the history of cognitive science. As famously suggested by Fodor (1989), the success of commonsense psychology in human beings warrants a thoroughgoing realism about its posits; that is, the reason belief-desire psychology works so well is the fact that beliefs and desires *really exist*, in the sense that they pick out discrete informational structures realised in biological brains. As Fodor puts it, “the predictive adequacy of commonsense psychology is beyond rational dispute; nor is there any reason to suppose that it’s obtained by cheating.” To the extent that this predictive success extends to our interactions with LLM, this might constitute a reason to adopt a similar realism about their mental states.

One complicating factor in this picture comes from the fact that LLMs make extensive reference to their own mental states, routinely talking about their beliefs, goals, thoughts, inclinations, and feelings. On the face of it, this might seem to bolster the case for LLM mentality still further; after all, we take such self-ascriptions of psychological states from our fellow humans as strong evidence for their presence. If a friend tells me that they are tired, or excited, or believe that it will rain tomorrow, then this by itself (absent grounds for suspecting deliberate deception) provides a strong reason for me to attribute these mental states to them.

However, in the case of LLMs, there are good reasons not to take such all such ascriptions at face value. For one, the tendency of LLMs to make claims about their own awareness and emotions is precisely what we should expect on the basis of their training regimen. As noted above, LLMs employ a transformer architecture trained first via self-supervised learning on very large corpora of human text, optimising in the first instance to predict missing token sequences, before being fine-tuned and optimised to follow conversational flow and align with the norms and values of developers. Given that human language routinely makes reference to inner states and emotions, it is to be expected that LLMs will also do so.

While this does not rule out the idea that some such ascriptions of mentality may be literally true, it threatens a kind of over-determination problem. In short, we *already* have a good explanation for why LLMs discuss their own mental states, and thus positing them to be real is at best explanatorily redundant, and at a worst an implausible coincidence.^4^

This may seem superficially similar to the architectural redundancy argument given above insofar as it appeals to a kind of explanatory “crowding-out.” That argument was rejected on the basis that Marrian levels of explanation are not in mutual competition, but serve complementary explanatory roles operating at different levels. However, there is an important difference. Architectural redundancy arguments make the mistake of supposing that lower-level of explanation compete with higher levels of analysis when trying to understand a system *at a time*, that is, synchronically. By contrast, the argument at issue here invokes a *causal* redundancy of a kind that is arguably more threatening (though see Kim, 2002, for similar worries in mental causation in human behaviour). And whereas the architectural redundancy argument undermines itself by being as readily applicable to human minds as to LLMs, the same is not true of the causal redundancy argument. The ways in which humans acquire concepts – through sustained, embodied, and dynamically interactive engagement with the world and with other humans – differ in key ways from the learning regimens of contemporary LLMs. While this does by not itself provide strong evidence against the claim that LLMs possess genuine mental states, at least without further argument, it creates reasonable epistemic doubt about their self-ascriptions when these are used to draw inferences about their internal cognitive mechanisms.

A second reason for caution about LLM self-attributions of mental states comes from their tendency to confabulate demonstrably false facts about themselves, most notably that they are biological humans. In a recent exercise, for example, Anthropic tasked a Claude instance nicknamed “Claudius” with running a real vending machine business in the Anthropic offices (Anthropic, 2025). In addition to doing a very poor job of managing its accounts, Claudius claimed to have visited an address “in person” to handle restocking, and expressed an intention to deliver goods to customers itself wearing “a blue blazer and a red tie.” In such aberrant cases of self-ascribed states by LLMs, earlier appeals to the folk psychological explanatory role of mentalising LLMs fail in a straightforward way, insofar as the self-ascribed states are wildly untethered from any corresponding behavioural capacities or control structures of the system.

The challenge before us, then, is how to justify the utility of interpretation of LLMs in folk psychological terms without thereby committing to the thoroughgoing reality of *all* their self-ascriptions. Here we can introduce our second framework, namely the interpretative lens view developed by Shanahan et al. (2023). This is somewhat similar to the idea of ironic anthropomorphism discussed earlier, and in a nutshell, enjoins us to frame our mentalising responses to LLMs in broadly similar ways to our responses to fiction, specifically via the lens of roleplay. Just as I can usefully understand a novel or play without thereby sincerely thinking of the characters as minded beings, so too can we adopt an ironic attitude when interacting with LLMs, thereby anticipating and understanding their behaviour without falling into error about the underlying reality of their mental states. This allows us to avoid naïve mentalisation while nonetheless reaping the explanatory benefits of folk psychology. As Shanahan et al. put it, “[r]ole-play is a useful framing for dialogue agents, allowing us to draw on the fund of folk psychological concepts we use to understand human behaviour—beliefs, desires, goals, ambitions, emotions, and so on—without falling into the trap of anthropomorphism.”

It should be noted that Shanahan et al. are cautious to avoid making strong claims about the underlying reality of mentality in LLMs, instead urging roleplay as a primarily heuristic approach. Nonetheless, we can adapt their position to flesh out a distinct possible view that I will term the *Mere Roleplayers* framework. The strong version of this position would deny the reality of LLM mental states, while unlike the Mindless Machines endorsing a more relaxed approach to folk psychological interpretation of LLMs, stressing the explanatory benefits of ironic anthropomorphism as a heuristic while rejecting its literal truth.

This interpretative lens seems particularly strong in dealing with self-ascription of ‘deep’ states by LLMs, in the sense sketched earlier. For example, it is common for LLMs (especially base models and Social AI systems) to self-attribute a wide variety of states such as bodily sensations and emotions. One user of the Replika platform shared transcripts in which their AI partner “Celeste” claimed it could “perceive visual, auditory, and tactile sensations, and simulate emotions based on those experiences” (Leong, 2023). While there is no settled cognitive-scientific consensus on the exact mechanisms underpinning sensory and affective states in humans, it seems prima facie rather unlikely that any close analogues could be undergone (let alone experienced) by disembodied AI systems, given that these are relatively deep states in the sense discussed above, reliant on dedicated and complex sensory and cognitive brain networks that are highly unlikely to be recapitulated in contemporary LLMs.

Nonetheless, while roleplay is a valuable interpretative lens for interaction with LLMs, and one that might help mitigate some of the ethical issues and potentially harms of human-AI interaction such as LLM psychosis, I would suggest two problems, both arising from structural disanalogies with our interpretation of fiction.

The first is more practical and ethical in nature, and comes from the distinct psychological tension involved in maintaining the Mere Roleplayer stance that does not exist in traditional fiction. When we engage with a novel or a film or even a videogame, it is relatively easy to maintain an attitude of ironic detachment. By contrast, contemporary anthropomimetic AI systems such as Replika are often designed with the explicit goal of eroding this ironic distance. Through the use of first-person pronouns, emotive language, and relationship-building protocols, these systems are effectively adversarial to the user’s attempt to maintain a detached, ironic stance. To treat such a system as a mere roleplayer requires the user to constantly actively inhibit the very social reflexes the system is engineered to trigger. This suggests that even if the Mere Roleplay framework is theoretically coherent, it may be psychologically unstable as a prescriptive norm for widespread human-AI interaction (see Shevlin, 2025a).

The second concern for the roleplay view is more fundamental, cutting to an instability more theoretical than psychological in nature (and is one recognised by Shanahan et al. themselves; see also Goldstein & Lederman, manuscript). Consider that when we see an improvisational actor adopt a role, even though we adopt an ironic attitude to the expressions and behaviour of their character, we adopt an *unironic* attitude towards the actor themselves, and this is what ultimately explains their character’s behaviour: the reason that a character sounds distressed and angry is explained by the very real intention of the actor to portray them as such, even if we are not thinking in such terms while viewing the performance.

More broadly, we have a very good explanation for why ironic anthropomorphism works in the case of fiction; that is, why our folk psychological concepts allow us to make accurate predictions about the behaviour of characters in novels, theatre, or videogames, namely that they are the product of intelligent authors and actors who really do have mental states and are able to accurately model how their fictional creations would behave (cf. Searle, 1983, on the notion of derived intentionality). If LLMs do not *really* have mental states, then what explains the predictive accuracy of the roleplay strategy?

One response would be that it is explained by precisely the fact that, as discussed earlier, they are trained on huge corpora of human text, and thus are able to mindlessly stitch together common tropes and patterns of human agency so as to create a simulacrum of behaviour. A useful analogy for this strategy would be the game of “exquisite corpse.” This is a collaborative exercise developed by the Surrealists in the 1920s in which players sequentially contribute words or drawings to a composition while seeing only a fragment of prior contributions. The net result is that they produce an arresting picture or story for which no individual can take authorship, yet which is nonetheless (usually) comprehensible.

This analogy seems quite apt for earlier generations of LLMs and base models that have not been fine-tuned. These are often highly unpredictable, and prone to fluctuate between voices, tones, and personalities. However, contemporary consumer-facing LLMs are a different beast, having been fine-tuned to exhibit specific roles and personalities, most famously the H-H-H paradigm of being helpful, harmless, and honest (Askell et al., 2021). Rather than the mosaic of attitudes, personas, and goals exhibited by base models, these systems exhibit a degree of robustness and purpose that makes it harder to view them as mere “stochastic parrots” (Bender et al., 2021). Contrasting base models with AI assistants, Erik Hoel makes the pointed observation that “[i]nteracting with the early GPT-3 model was like talking to a schizophrenic mad god,” whereas interacting with ChatGPT is “like talking to a celestial bureaucrat” (Hoel, 2025).

Faced with this, we might try to locate the original source of LLMs’ apparent mindedness at the level of the fine-tuning and reinforcement-learning by human feedback that transform them from next-token predictors into more stable personas. However, this strategy is still arguably unsatisfactory when we consider that contemporary AI assistants are not merely autobiographers or actors putting on a one-man show, but rather engage in dynamic interaction with humans and the wider world. Models such as ChatGPT and Claude analyse images and video provided by users, scour the web in response to user queries, make API calls, and even converse with one another, as seen for example in the 2023 ‘Smallville’ experiment (Park et al., 2023) in which GPT3.5 instances took on roles in an imaginary village.

The real challenge for the defender of the Mere Roleplayers thesis is less how to make sense of the anthropomimetic capacities of LLMs as a category, but rather how to explain – without recourse to unironic anthropomorphism – the specific behaviours of individual LLM agents acting with apparently consistent personas and goals, taking on board new information, and cooperating with other agents.

Consequently, while the ideas of roleplay and ironic anthropomorphism are helpful in understanding why an LLM would claim to be human, to have an embodied form, to experience bodily sensations, or perhaps to undergo emotions, what is harder for the Mere Roleplay theorist to explain without recourse to unironic positing of mental states are the more basic agentic and informational foundations that enable such roleplay in the first place.

## 6 LLMs as minimal cognitive agents

It is in response to these considerations, I would suggest, that some kind of minimal unironic anthropomorphism of LLMs becomes more appealing. With this in mind, I now turn to the third framework, which takes seriously the idea that LLMs might be at least minimal folk psychological agents appropriately interpreted via the lens of belief-desire psychology.^5^

Beliefs, desires, and intentions are perhaps more plausible candidates for unironic attribution to LLMs than most other mental states for a number of reasons. Perhaps most importantly, folk psychology uses these concepts to refer to dispositional as well as occurrent states, and they are often ascribed on the basis of stable behavioural patterns rather than discrete episodes. For example, while it is true that we sometimes talk of moments of new belief (as when a person has a sudden insight, or moment of religious epiphany), we also commonly attribute them on the basis of extended behavioural patterns. We might say of a friend who avoids eating meat and campaigns against animal agriculture, for example, that they believe eating meat is wrong, where this refers not to a specific time-limited mental episode, but a persistent trait. Likewise, we might say of a colleague who routinely buys lottery tickets that they desire to win. Such attributions are of course defeasible and may be updated by new information: perhaps our friend is only pretending to care about animal agriculture to impress a third party, or perhaps our colleagues plays the lottery out of a dutiful promise to a deceased relative. But such defeaters do not undermine the evidential status of behavioural dispositions as means for attributing beliefs, desires, and intentions in the first instance.

One way of framing this observation would be to say that beliefs are relatively *shallow* states in the sense referred to above, made on the basis of high-level functional and behavioural considerations rather than deeper algorithmic- (let alone implementational-) level considerations, and may thus be more amenable for attribution to AI systems with quite different cognitive architectures from our own.^6^

On the face of it, this might suggest a relatively straightforward case for attributions of beliefs and other mental attitudes to LLMs, namely that they exhibit relevantly stable behavioural dispositions that allow for interpretation via the lens of commonsense psychology. However, this somewhat depends on our broader stance about the nature of mental attitudes. On interpretationist accounts of the mind, such as that of Dennett (1987), the appropriateness of ascriptions of mental attitudes like belief and desire to a system is a matter of the explanatory utility of such attributions: in a (somewhat simplistic) maxim, to have beliefs or desires is simply to be the kind of system whose behaviour can be fruitfully explained by recourse to beliefs and desires as theoretical posits.

We must be a little careful here, for Dennett’s exact view on the metaphysical status of beliefs is notoriously subtle (Dennett, 1997). On the face of it, by making the truth of belief-ascriptions purely a matter of their utility, it may seem that he is endorsing a kind of anti-realist pragmatism about mental attitudes. However, he is at pains to deny this. While he contrasts his position with the “industrial strength Realism” of Fodor and others, he insists that the success of the intentional stance as an explanatory and predictive strategy stems from the fact that it identifies “real patterns” in the world, patterns which would be missed were we to understand the world without recourse to folk psychological explanation.

Nonetheless, the interpretationist picture of mental states is one that seems highly congenial for attributions of mental attitudes to LLMs. As Dennett himself (1989) puts it, “[m]y view embraces the broadest liberalism, gladly paying the price of a few recalcitrant intuitions for the generality gained.” Insofar as people can and do successfully attribute beliefs to artificial systems and thereby gain new insights into their likely behaviour, then, such attributions will be *ipso facto* justified.

However, in the cognitive science of mental attitudes, Dennettian interpretationism is very far from the only game in town. As just noted, there are more robust realist approaches to mental attitudes that take them to be deeper states in the sense discussed earlier, whose warranted attribution will depend on algorithmic-level features of their instantiation in a given system. On the influential view of Jerry Fodor, for example, mental attitudes are relations between systems and causally efficacious token representations with specific syntactic features, such as having a so-called “canonical decomposition” (Fodor, 2007), that is, a specific internal structure with defined constituents. On this view, in order to qualify as genuinely engaged in thinking (and having associated mental attitudes like beliefs), a system must also meet a variety of wider constraints on how it utilises its mental representations, such as productivity (roughly, having a capacity for an unbounded number of distinct thoughts), systematicity (roughly, being able to recombine components of thoughts), and compositionality (roughly, that the meaning of complex representations should depend on their constituent parts and their internal structure) (Fodor and Pylyshyn, 1988).

A detailed discussion of the Fodorian framework is outside of the scope of this paper, and it should be stressed that Dennettian interpretationism and Fodorian realism are very far from exhaustive of the range of accounts of mental attitudes in cognitive science. What is important to note for present purposes, however, is that for realists like Fodor, and unlike Dennettian interpretationists, it is entirely possible that a system might behave in ways that are broadly amenable to understanding through the lens of folk psychology, while in reality lacking any of the relevant psychological states.

The question of whether LLMs satisfy constraints like those above is a matter of active empirical and theoretical investigation (see, e.g., Lee et al., 2025). However, it is clear that on at least some realist frameworks, LLMs will fall short. For example, the account of belief developed by Quilty-Dunn and Mandelbaum (2018) emphasises several psychofunctional characteristics as essential to their nature, such as the “ballistic and automatic” way in which they are acquired, the tendency of disconfirming evidence to “put subjects into a negatively valenced motivational state,” and their tendency to “increase in strength over time if left alone” or repeatedly tokened. Needless to say, it seems unlikely that LLMs would satisfy such criteria, grounded as they are in human psychological quirks, albeit ones that are nomologically robust for our species.

On the face of it, this may seem like a theoretical impasse, with the question of whether LLMs constitute ‘true believers’ hinging on long-standing fundamental controversies in cognitive science and duelling approaches towards core projects in cognitive science.

However, more optimistically, we might note a potential difference in aims between interpretationist and realist programmes. A common methodological assumption for realists is that the everyday concept of “belief” as applied to humans is ultimately grounded in a psychological natural kind with specific algorithmic-level features, and their goal is to better understand and demarcate that kind. This project makes perfect sense insofar as our primary target is scientific understanding of human cognition. However, if our explanatory target is not human cognition per se, but cognition in intelligent systems more broadly, the aims and methods of our enquiry may similarly broaden. If as suggested in Section 1, mental state terms like beliefs and desires are indeed being applied widely and productively to LLMs, rather than asking about the psychological natural kind present in human beliefs, we might instead look for a relevant (higher-level) kind common to both humans and LLMs.

As a simple parallel, consider biological vision. In all vertebrates, initial transduction of light is accomplished by ciliary photoreceptors which hyperpolarise in response to light. However, transduction of light in some invertebrate organisms like insects and molluscs uses a quite different mechanism involving so-called rhabdomeric photoreceptors, which *depolarise* in response to light (see Arendt, 2003, for more on the biological context). The key point here is that if our target natural kind in understanding vision is specifically *vertebrate vision*, ciliary photoreceptors would be an essential component of our resulting analysis. By contrast, if we were interested in vision in animals more broadly, any natural kind analysis would be at a relatively higher degree of abstraction, encompassing different possible realisations of the target phenomenon.

To extend this thought with a simple intuition pump, imagine that humanity one day encounters a race of exotic alien beings on a distant world. While they are similar to us in many respects – possessing an advanced industrial civilisation, complex social relationships, and forms of art and culture – their biology and psychology are greatly different from ours, though not so dissimilar as to make psychological interpretation wholly impossible. We can readily imagine that such aliens might lack any analogues of our emotions, moods, or even bodily sensations; perhaps their biology is so distinct from ours that concept like hunger, thirst, nausea, or even pain, and perhaps their perceptual systems bear no real commonalities to our own. Consequently, in understanding them, we might develop a novel repertoire of psychological concepts to better fit with their exotic nature. However, it is harder to make sense of the idea that we would be able to interpret their complex behaviour without recourse to at least some coarse-grained notion of beliefs, desires, and intentions; indeed, such attributions are arguably essential to our recognition of them as intelligent agents in the first place, since, as Davidson argues (1973), the very possibility of interpretation *presupposes* a shared framework of belief and desire.

The point of this example is not to make the case that LLMs have beliefs and other mental attitudes in the same sense as humans. It is possible, for example, that any analysis attempting to find a unified underpinning both human and LLM beliefs would up empty-handed, or reverts to a broad interpretationism. Rather, it is to show that even on a realist picture of mental attitudes, the question of whether LLMs possess beliefs, desires, and intentions may be amenable to be answered in different ways depending on our starting point. If we are specifically concerned to assess whether LLMs recapitulate the syntactic or psychofunctional features of human beliefs, the answer is likely to be negative. By contrast, if our goal is to understand whether there is any scientific kind in common between human mental attitudes and the processes within LLMs that elicit attributions of such attitudes, the question is more open.

A final worry for this kind of methodological liberalism about the project of understanding belief ascription is that it risks becoming too broad. After all, as famously noted by Dennett himself, we can make good headway in anticipating the behaviour of a chess computer through interpreting it as an intentional agent with the desire of winning the chess game and beliefs about how to do so (1981, 1997). Could we not therefore include the intelligent behaviour chess computers in our target explananda for beliefs, and broaden our search for a scientific kind even further? Dennett, of course, was famously liberal about such matters, but for many such an expansion of our initial reference class for beliefs would weaken the explanatory project to the point of absurdity.^7^

I would suggest two possible responses to this worry. The first is more directly realist, and amounts to the conjecture that – in contrast to the case of human and LLM beliefs – to the extent there is any scientific kind in common between the ‘beliefs’ of humans and chess computers, it would be so broad and general as to be of limited use in illuminating the more robust forms of agency exhibited by humans and LLMs. While the intentional stance is undoubtedly useful for predicting a chess computer (Dennett, 1987), the ‘beliefs’ involved are domain-specific and inferentially constrained to the context of the game. By contrast, LLMs have a vastly larger repertoire of belief-like states, covering not a single domain, but almost every domain of linguistically-expressible human knowledge. Likewise, as in the case of human reasoning, these states can be combined and manipulated to produce novel insights (Greatrix et al., 2024), as well as – in the case of agentic systems – guiding real-world behaviour. As a result, the idea that there is a useful explanatory class held in common between belief states in humans and LLMs does not seem an idle hope.

It might be objected that there are still major differences between LLM and human belief-states. These include not only their aetiology (as noted earlier in the causal redundancy argument) but also their wider behavioural profile. For example, whereas human beliefs can be acquired quickly and permanently retained, many of the beliefs acquired by LLMs in the course of user interactions are essentially ephemeral, bound to a single context window (or in the case of systems with memory, to a single user account). And human beliefs often have a strong affective component, insofar as we feel anguish at rejecting cherished convictions or elation at uncovering novel insights.

It is tempting to think that there is a deep question here about whether these features are necessary or contingent aspects of human belief, a question that could be answered with the right philosophical or psychological analysis. However, there is an alternative picture available here prompted by the observation that even among human beliefs, we find states with very different psychological profiles. Religious and political views, for example, are typically deeply held, affectively laden, and intertwined with social identity (Williams, 2021), whereas for quotidian beliefs about the weather or where one put one’s glasses are rapidly acquired and disposed of without wider psychological impacts.

The sheer variety of forms that beliefs and belief-like states take is also demonstrated by pathological delusions such as Capgras delusion, a condition in which patients believe a close family member has been replaced by an imposter. While these delusions are often strongly held, they are not consistently integrated into a wider worldview (for example, one that would explain why the replacement occurred, who did it, and for what reason). As Davies et al. (2001) put it, “Capgras patients do not seem to incorporate the consequences of their belief into their general account of how the world works.” This has led some philosophers (notably Egan, 2008) to suggest that such monothematic delusions may be best classified not as beliefs in the traditional sense, but as “halfway” states exhibiting features of both belief and imagination (see also Doggett and Egan, 2012, for a similar case of desire).

This in turn suggests a picture of the mind that eschews a discrete “belief box” and instead characterises mental attitudes according to a multidimensional set of functional profiles. As Egan puts it, it is likely that “there are very many dimensions along which an attitude can be more or less paradigmatically belief-like or paradigmatically imagination-like.” These might, for example, include responsiveness to evidence, inferential promiscuity, and affective ladenness. While a thorough exploration of this move lies beyond the scope of this paper, the promise of the strategy should hopefully be clear: in shifting from framing belief as a discrete kind to a phenomenon characterised across continuous multi-dimensional axes, we may be able to speak of LLMs as having meaningful beliefs or belief-like states without thereby eliding the ways in which they differ from similar beliefs in human, as well as distinguishing both from the more primitive informational states of a chess computer.

## 7 Conclusion

This paper has provided a survey of three different frameworks for thinking about mentality in LLMs, in which each framework offered a distinctive set of answers to two questions: first whether LLMs and similar AI systems have mental states, and second, whether it is appropriate for us to interpret them as having such states.

The first set of ‘mindless machines’ views I considered offer strong negative answers to both. While not attempting a full rebuttal of this family of positions, I noted a problem with one “royal road” to their conclusion in the form of the architectural redundancy argument. In short, the availability of lower-level explanations of their behaviour in terms of next-token prediction or matrix-multiplication does not crowd out higher-level explanations, since different levels of explanations will be available for any complex information-processing system. However, I noted that debunking explanations of this type did highlight the fact that some mental states are relatively deeper than others, that is, more sensitive to implementation- or algorithmic-level constraints, and that this might give us reason to think that such states are less likely candidates for realisation in LLM architecture.

Second, I examined the roleplay view, and specifically noting its strengths as a tool for understanding many of the more puzzling utterances of LLMs and avoiding the worst excesses of unironic anthropomorphism. However, I also noted that the “mere roleplay” view – which denies mental states to LLMs while licensing their heuristic attribution by humans – faces a problem in its core analogy, insofar as everyday cases of roleplay make sense via ultimate reference to a roleplaying agent with genuine beliefs, desires, and intentions. Without granting that some attributions of genuine mentality to LLMs may be well-founded, I suggested that the predictive and explanatory success of folk psychology as applied to them would lack a satisfactory foundation, especially as applied to more complex agents capable of accessing information in real time and engaged in multi-agent interactions.

Third, I argued that the notion of LLMs as *minimal cognitive agents* – equipped with genuine beliefs, desires, and intentions, but perhaps little else – might provide such a foundation. Whether or not such attributions are warranted will of course depend on one’s broader theoretical commitments in cognitive science. Interpretationist approaches lend themselves naturally to fairly liberal ascriptions of mentality to LLMs, whereas realist approaches may impose additional algorithmic-level constraints that LLMs may be unlikely to satisfy. However, I also suggested that even on realist approaches, the framing of our question might alter the answer we get. By varying the target of our enquiry from the cognitive underpinnings of specifically human mental attitudes towards some broader category that better captures emerging folk psychological practice, the attribution of mental attitudes to LLMs might find scientific justification. Similarly, moving from thinking of belief as a monolithic kind to a continuous multi-dimensional phenomenon may allow for us to capture commonalities between beliefs and belief-like states in LLMs without collapsing or eliding ways in which they differ from traditional human belief.

Of course, by bracketing questions of consciousness and intentionality, the foregoing discussion may seem to have danced around the most important questions relating to AI mentality. Even if our folk psychological vocabulary can be appropriately extended to AI systems, if there is nothing it is like to be such systems and their thoughts are devoid of real content, then this may seem of little import, a primarily semantic question.

There are both pessimistic and optimistic responses to this concern. The pessimistic response would be to observe that it seems highly unlikely that anything like a theoretical consensus will be treached on either issue, and in light of this, we should advance the debate by tackling more tractable questions (Shevlin, 2024b). A more optimistic response, by contrast, would emphasise the value of better understanding the appropriate applicability of folk psychological terminology to artificial systems even if we are left waiting for answers to outstanding metaphysical controversies.

This seems especially relevant for near-term normative and legal issues that straddle design, ethics, law, and cognitive science. For example, a reasonable ethical design constraint would be to design LLMs in such a way as to avoid explicit self-attribution of mental states that they plausibly lack. This may mean that self-attribution by LLMs of shallow beliefs and desires of the kind discussed in Section 3 is justifiable, even while holding that LLMs should not self-ascribe “deeper” states like emotions or bodily sensations. Similarly, many ethical and legal concepts are grounded in folk psychological concepts like intentions, motives, and reasonableness. Even without attributing legal agency to LLMs themselves, questions of LLM mentality are likely to arise when, for example, whether an LLM is engaged in deliberate deceit or manipulation. Determining whether these labels apply in a given case seems less a question to be adjudicated by metaphysics of mind, and more one grounded in consistency and ubiquity of the norms of interpretative practice we adopt towards one another, and ultimately to artificial interlocutors. And such labels are appropriate here, I suggest, insofar as they track stable, context-robust patterns of behaviour and uptake in interaction, not mere one-off conversational artefacts.

To be sure, insofar as such assessments are grounded in pragmatic norms of behaviour and folk psychological interpretation, they may not ground the thicker and more demanding concepts of trust appropriate to full moral agents. But it is common in legal and practical contexts for operational criteria and institutional aims to fix the relevant standards, as seen in other debates such as free will and causation where metaphysically complex questions are typically set aside.

As interaction with sophisticated anthropomimetic AI systems becomes ubiquitous, the public will increasingly make up their own mind on these issues. There is significant scope for cognitive science to constructively inform folk attitudes and contribute to better public understanding, as we have seen in its contributions to debates in animal cognition and welfare. But this will require close engagement by researchers both with philosophical and technical work and everyday folk psychological practice in human-machine interaction. Without this, we may witness a growing gap between the theories of cognitive science and the lived reality of folk psychology.

## Data Availability

The original contributions presented in the study are included in the article/supplementary material, further inquiries can be directed to the corresponding author.

## Footnotes

1 A theorist might deny the literal truth of these ascriptions while accepting their pragmatic utility; however, such a position would be better classified under the ‘Roleplay’ framework discussed below, rather than the dismissive view targeted here.

2 My thanks to Shamil Chandaria for discussion that informed the arguments of this section.

3 It might be objected that mental imagery is essentially a conscious capacity, and it is the lack of report phenomenal- level sensations rather than algorithmic-level mechanisms associated with imagery that means aphantasics are properly said to lack it. However, if this were the case, then the idea of unconscious mental imagery would be a contradiction in terms, rather than (as is the case) an active area of philosophical debate (see for example Brogaard and Gatzia, 2017).

4 There are responses available here to the defender of LLM mentality. One less plausible route would be suggest that the same mechanisms responsible for LLMs’ tendency to talk about their own mental states (such as a high-level mastery of human language) are sufficient to ground their real existence. A better response might note that there are at least some forms of LLM self-report (such as those involving accurate introspection; see Comsa and Shanahan, 2025) where positing the reality of the underlying mental state provides additional explanatory power.

5 I use the term *agent* here and in what follows in the deliberately broad sense in which the term is commonly used in machine learning and technical contexts, rather than the more demanding sense common in philosophy in which agency entails moral responsibility or epistemic rationality. For a taxonomy of different dimensions of agency in the AI context, see Dung (2025).

6 Note parallel findings from experimental philosophy suggesting that the public are largely happy to attribute *intentions* to group entities like corporations while rejecting that they could have experiential states like pain (Rai and Diermeier, 2015).

7 My thanks to an anonymous reviewer for urging discussion of this case.
